# Multifunctional Detection Sensor and Sensitivity Improvement of a Double Solenoid Coil Sensor

**DOI:** 10.3390/mi10060377

**Published:** 2019-06-05

**Authors:** Laihao Ma, Zhiwei Xu, Hongpeng Zhang, Weiliang Qiao, Haiquan Chen

**Affiliations:** Marine Engineering College, Dalian Maritime University, Dalian 116026, China; malaihao@dlmu.edu.cn (L.M.); xuzhiwei123@dlmu.edu.cn (Z.X.); chenapec@163.com (H.C.)

**Keywords:** oil contaminant, solenoid coil, multifunctional sensor, microfluidic chip

## Abstract

A multifunction detection sensor for hydraulic oil contaminants based on a microfluidic chip is proposed, which consists of double solenoid coils and a straight microchannel. The inductance detection model of metal particles and capacitance detection model of nonmetal particles are constructed theoretically. In order to further improve detection sensitivity, experiments of effects of silicon steel sheets on the sensitivity of detection are carried out. Experimental results show that the silicon steel sheets can significantly improve the detection sensitivity of metal particles. The inductance amplitude and signal-to-noise (SNR) of iron particles ranging from 60–130 μm and copper particles ranging from 120–180 μm can be increased by at least 7.0–2.4 and 4.5–2.0 times, respectively. We demonstrate the successful detection of 30 μm iron particles and 90 μm copper particles using double solenoid coils with silicon steel sheets. In capacitance detection experiments, the silicon steel sheets can improve the sensitivity of capacitance detection, but the improvement effect is not obvious. We demonstrate the successful detection of 140 μm water droplets and 240 μm bubbles using double solenoid coils with and without silicon steel sheets. The capacitance amplitude and SNR of detecting water droplets ranging from 140–150 μm and bubbles ranging from 240–250 μm can be increased by 37.4–21.9% and 18.5–8.0% using double solenoid coils with silicon steel sheets, respectively.

## 1. Introduction

Oil contamination has become one of the most important causes of wear and failure of hydraulic components [1,2]. Oil contaminants mainly include water, air bubbles, and metal particles, each of which has different hazards to the hydraulic system [3,4]. The mixing of water droplets in hydraulic oil will increase the emulsification rate of oil [5]. The air mixed in hydraulic oil will generate cavitation, accompanied by an increase in vibration and noise [6]. The size of metal wear debris in hydraulic oil can reflect the degree of wear of hydraulic components. The metal wear debris in oil is about 10–20 μm in size under normal conditions, and it will increase to 50~100 μm in size when abnormal wear occurs [7]. The properties of metal wear debris can also reflect the wear position of hydraulic components. In addition to the use of ferromagnetic metals in hydraulic component manufacturing, the friction pairs of hydraulic components are often coated with a layer of nonferromagnetic metals to reduce friction, such as a sliding shoe, a cylinder block, an oil distribution plate, etc., in the axial piston pump and are mostly made of copper alloy. Therefore, the realization of on-line discrimination detection of oil contaminants can provide technical support for health status monitoring and fault diagnosis of hydraulic systems.

Oil contaminant detection technology is mainly divided into off-line detection and on-line detection [8]. It is difficult for off-line detection to reflect the actual oil physical and chemical properties of the hydraulic system during operation, as it is subject to many sampling procedures and low timeliness in the laboratory [9,10]. At present, oil on-line detection technology includes ultrasonic detection, optical detection, capacitance detection, and inductance detection. Although ultrasonic detection can distinguish between metal particles and bubbles, it cannot distinguish between ferromagnetic metal particles and nonferromagnetic metal particles, and it is susceptible to external environments such as oil temperature and mechanical equipment noise [11,12]. Although optical detection has high detection accuracy, it is easily affected by oil clarity and permeability, and it is impossible to distinguish solid particles [13,14]. Capacitance detection can distinguish between water droplets and air bubbles because of different permittivity but is insensitive to detecting metal particles [15,16,17,18,19]. Inductance detection has high linearity in distinguishing ferromagnetic and nonferromagnetic metal particles. Flanagan et al. conducted oil particle detections with a coil 6 mm length and successfully detected 100 μm iron particles and 200 μm copper particles [20]. Hongzhi et al. designed a metal particle detection sensor with three solenoid coils, which could detect particles with a size of 125 μm [21]. Hongbo et al. designed a double-coil metal particle detection sensor, which realized the detection of 100 μm iron particles and 500 μm copper particles [22]. The low-detection sensitivity of inductance sensors mentioned above is due to the low magnetic field intensity of coil in the large flow channel. The application of microfluidic technology in oil detection improves the sensitivity of inductance detection. Du et al. realized on-line differential detection of iron and copper particles ranging in size from 50 to 150 mm using a two-layer planar coil with amplification circuit and noise shielding techniques [23], and they detected 32 μm iron particles and 75 μm copper particles by parallel connection of inductance coil and capacitor [24]. However, these methods for detection sensitivity improvement require the aid of an external circuit or capacitor, which makes the fabrication process more complex. Zhu et al. developed a high-sensitivity wear debris sensor with two planar coils wound around a pair of ferrite cores that could detect 50 µm ferrous debris in 7 mm diameter fluidic pipes but not droplets and bubbles in oil [25]. Our group designed a sensor integrated with inductance and capacitance detection using double planar coils, which can distinguish iron particles, copper particles, water droplets, and bubbles in oil, but the detection sensitivity needs to be improved [26,27,28].

As a planar coil with many turns, the outer coil turns of the planar coil are not effective in enhancing the magnetic flux density at the sensing zone [23]. In order to improve the sensitivity of multiparameter detection of oil contaminants, we developed in this paper an impedance sensor with double solenoid coils that had the functions of inductance and capacitance detection. Detection sensitivity was further improved by embedding silicon steel sheets in the solenoid coils.

## 2. Chip Design and Detection Model

### 2.1. Chip Design

**Figure 1:**
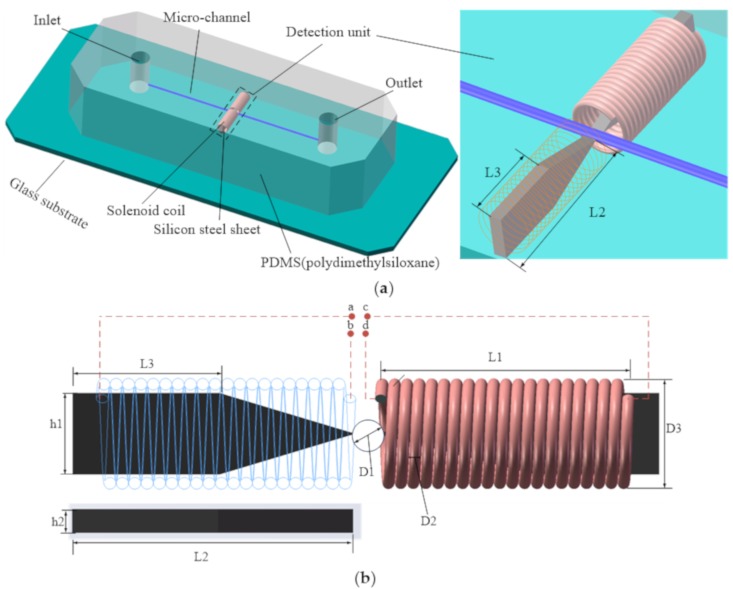
The design of the multifunction sensor: (**a**) the overall design of the chip, and (**b**) a sketch of the detection unit.

The multifunction detection chip design is shown in Figure 1. The chip was mainly composed of a straight microchannel and a pair of solenoid coils on both sides of straight microchannel. The diameter of microchannel D1 was 300 μm. The diameter of solenoid coil wire core D2 was 70 μm (an insulating paint with a thickness of 10 μm covered the core). The diameter of solenoid coil outer hole D3 was 1.9 mm. The axial length of solenoid coil L1 was 2.8 mm. In order to focus the magnetic field strength and the electric field intensity generated by solenoid coils in the microchannel detection region, a silicon steel sheet was inserted into each of the two solenoid coils (see Figure 1b—as the shape of silicon steel sheet is tapered, L2 is the length of the whole silicon steel sheet, L3 is the length of the rectangular part of silicon steel sheet, h1 is the width of rectangular part of silicon steel sheet, and h2 is the thickness of the whole silicon steel sheet. L2 = 3.2 mm, L3 = 1.8 mm, h1 = 0.6 mm, and h2 = 0.1 mm). The inductance detection function and the capacitance detection function can be realized by changing the connection manner of the two solenoid coil leads. When the lead wires a and d of the solenoid coils were connected, the lead wires b and c were connected; that is, the two solenoid coils were connected in series, and thereby the inductance detecting mode could be realized. When the lead wires a and b of the solenoid coil were connected, the lead wires c and d were connected; that is, the two solenoid coils were respectively used as two plates, and thereby the capacitance detecting mode could be realized.

### 2.2. Inductance Detection Mode

**Figure 2:**
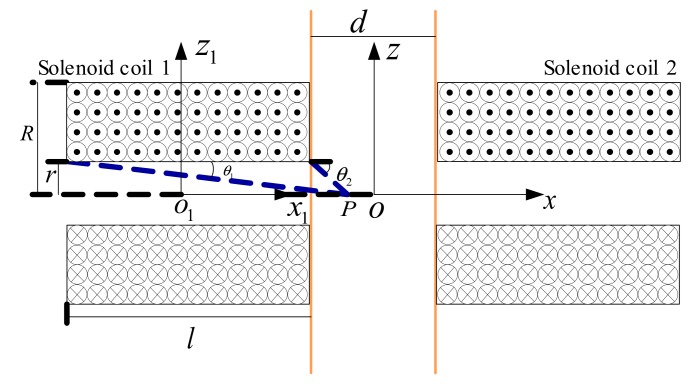
Two-dimensional coordinate system of double solenoid coils.

As the double solenoid coils were the same, it was assumed that the axial length of each solenoid coil was *l*, the number of turns was *n*, the inner diameter and outer diameter were *r* and *R*, respectively, and the number of solenoid coil layers per unit thickness was *a*. The midpoint *o*_1_ of the axis of the solenoid coil 1 and the midpoint *o* of the midline of the microchannel detection area were respectively taken to establish a two-dimensional coordinate system as shown in Figure 2.

For a single-layer solenoid coil in the coordinate system *o*_1_*x*_1_*z*_1_, the magnetic induction intensity of any point *P* on the central axis of the single-layer solenoid coil can be obtained according to Biot–Savar’s law [29]:

(1) $$B=\frac{\mu_{0}}{2}nI\left[\frac{x_{1}+\frac{l}{2}}{\sqrt{r_{{}^{1}}^{2}+{\left(x_{1}+\frac{l}{2}\right)}^{2}}}-\frac{x_{1}-\frac{l}{2}}{\sqrt{r_{{}^{1}}^{2}+{\left(x_{1}-\frac{l}{2}\right)}^{2}}}\right]$$

where $\mu_{0}$ is the magnetic permeability of the vacuum, and *I* is the coil current. Since the double solenoid coils used were multilayer solenoid coils, the axial magnetic induction intensity of the coil could be regarded as a superposition of the magnetic induction intensity of single-layer solenoid coil on the axis. As the thickness of the solenoid coil was *R − r*, the magnetic induction intensity of the point *P* of solenoid coil 1 could be obtained as follows by integrating Equation (1) along the thickness of the coil:

(2) $$B_{1}=\frac{\mu_{0}anI}{2}\left[(x_{1}+\frac{l}{2})\mathrm{In}\frac{R+\sqrt{R^{2}+{\left(x_{1}+\frac{l}{2}\right)}^{2}}}{r+\sqrt{r^{2}+{\left(x_{1}+\frac{l}{2}\right)}^{2}}}\right.\left.-(x_{1}-\frac{l}{2})\mathrm{In}\frac{R+\sqrt{R^{2}+{\left(x_{1}-\frac{l}{2}\right)}^{2}}}{r+\sqrt{r^{2}+{\left(x_{1}-\frac{l}{2}\right)}^{2}}}\right].$$

For ease of calculation, the coordinate system *o*_1_*x*_1_*z*_1_ was translated to the coordinate system *oxz*, and its translation relationship is:

(3) $$x_{1}=x-\frac{l}{2}-\frac{d}{2}.$$

Substituting Equation (3) into Equation (2), the expression of the magnetic induction intensity of point *P* in the coordinate system *oxz* of solenoid coil 1 is:

(4) $$B_{1}=\frac{\mu_{0}anI}{2}\left[(x-\frac{d}{2})\mathrm{In}\frac{R+\sqrt{R^{2}+{\left(x-\frac{d}{2}\right)}^{2}}}{r+\sqrt{r^{2}+{\left(x-\frac{d}{2}\right)}^{2}}}\right.\left.-(x-l-\frac{d}{2})\mathrm{In}\frac{R+\sqrt{R^{2}+{\left(x-l-\frac{d}{2}\right)}^{2}}}{r+\sqrt{r^{2}+{\left(x-l-\frac{d}{2}\right)}^{2}}}\right].$$

Similarly, expression of the magnetic induction intensity of point *P* in the coordinate system *oxz* of solenoid coil 2 is:

(5) $$B_{2}=\frac{\mu_{0}anI}{2}\left[(x+l+\frac{d}{2})\mathrm{In}\frac{R+\sqrt{R^{2}+{\left(x+l+\frac{d}{2}\right)}^{2}}}{r+\sqrt{r^{2}+{\left(x+l+\frac{d}{2}\right)}^{2}}}\right.\left.-(x+\frac{d}{2})\mathrm{In}\frac{R+\sqrt{R^{2}+{\left(x+\frac{d}{2}\right)}^{2}}}{r+\sqrt{r^{2}+{\left(x+\frac{d}{2}\right)}^{2}}}\right].$$

For Equations (4) and (5), taking *x* = 0, the magnetic induction intensities of solenoid coils 1 and 2 at the midpoint of the axis of microchannel detection area can be obtained:

(6) $$B_{1}=B_{2}=\frac{\mu_{0}anI}{2}\left[(l+\frac{d}{2})\mathrm{In}\frac{R+\sqrt{R^{2}+{\left(l+\frac{d}{2}\right)}^{2}}}{r+\sqrt{r^{2}+{\left(l+\frac{d}{2}\right)}^{2}}}\right.\left.-\frac{d}{2}\mathrm{In}\frac{R+\sqrt{R^{2}+{\left(\frac{d}{2}\right)}^{2}}}{r+\sqrt{r^{2}+{\left(\frac{d}{2}\right)}^{2}}}\right].$$

Since the directions of magnetic induction intensities of solenoid coils 1 and 2 were the same, the total magnetic induction intensity of double solenoid coils in the detection area is:

(7) $$B=B_{1}+B_{2}.$$

Combining with the definition of coil inductance, the total self-inductance of double solenoid coils in the detection area is as follows:

(8) $$L=L_{1}+L_{2}=\frac{n(B_{1}+B_{2})S}{I},$$

where S is the cross-sectional area of the inner hole of solenoid coil, and *S* = π*r*^2^. According to the definition of mutual inductance, the mutual inductance of double solenoid coils is:

(9) $$M=\frac{nB_{1}S}{I}=\frac{nB_{2}S}{I}.$$

Since the double solenoid coils are connected in series, an equivalent inductance can be obtained according to Kirchhoff’s law as follows:

(10) $$L_{eq}=L_{1}+L_{2}+2M.$$

The equivalent inductance of double solenoid coils in the detection area was obtained by combining Equations (4)–(10):

(11) $$L_{eq}=2\mu_{0}an^{2}\pi r^{2}\left[(l+\frac{d}{2})\mathrm{In}\frac{R+\sqrt{R^{2}+{\left(l+\frac{d}{2}\right)}^{2}}}{r+\sqrt{r^{2}+{\left(l+\frac{d}{2}\right)}^{2}}}\right.\left.-\frac{d}{2}\mathrm{In}\frac{R+\sqrt{R^{2}+{\left(\frac{d}{2}\right)}^{2}}}{r+\sqrt{r^{2}+{\left(\frac{d}{2}\right)}^{2}}}\right].$$

From Equation (11), the inductance variation was directly related to the relative magnetic permeability of the detected particles when the sizes of solenoid coils and the microchannel were determinate. When a ferromagnetic particle passed through the detection area, its magnetization played a major role; thus, the magnetic induction intensity of the coil and the coil inductance will increase. In contrast, there was almost no magnetization effect for a nonferromagnetic metal particle passing through the detection area; its eddy current would reduce the magnetic flux, which in turn would decrease the inductance of the coil. Therefore, ferromagnetic and nonferromagnetic metal particles can be distinguished by the directions of inductance pulse signals. At the same time, the size of metal particles can be judged by the height of inductance pulse signals. Compared with Equation (8), it can also be found that the inductance variation of metal particles passing through the detection area of double solenoid coils can be greatly increased compared to that of a single solenoid coil.

### 2.3. Capacitance Detection Mode

**Figure 3:**
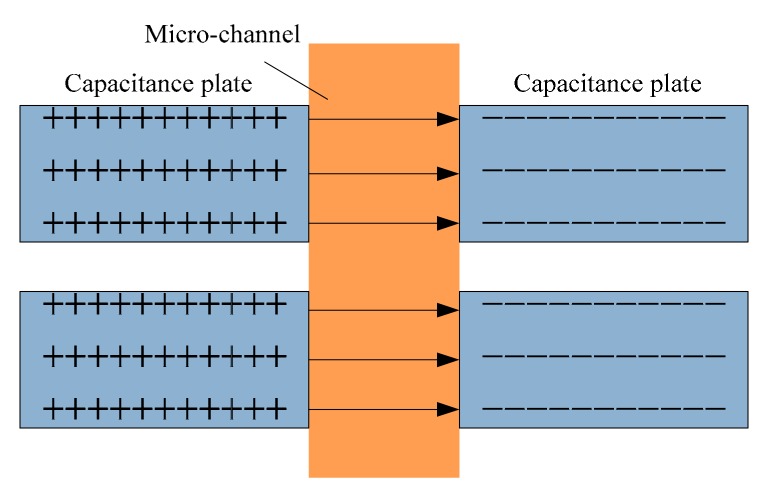
Schematic diagram of capacitance detection.

By changing the connection manner of double solenoid coils, the two solenoid coils can be regarded as the two plates, which can be equivalent to the cylindrical flat plate capacitor, as shown in Figure 3.

According to the calculation formula of a plate capacitor [30], the capacitance expression of an equivalent capacitor can be obtained:

(12) $$C=\frac{Q}{U}=\frac{\varepsilon_{0}\varepsilon_{r}S}{d}=\frac{\varepsilon_{0}\varepsilon_{r}\pi (R^{2}-r^{2})}{r},$$

where $\varepsilon_{0}$ is the vacuum permittivity, $\varepsilon_{r}$ is the relative permittivity of the media, and *S* is the plate area of the equivalent capacitance plate. 

From Equation (12), we know that when the arrangement of the double solenoid coils and microchannel is determinate, the capacitance is directly related to the permittivity of media in the detection zone. As the excitation source was a high-frequency, alternating current, the permittivity of the medium was also related to the excitation frequency. Therefore, the complex permittivity of a material is introduced:

(13) $$\tilde{\varepsilon }=\varepsilon -j\frac{\sigma }{2\pi f},$$

where ε is the permittivity, σ is the conductivity, *j*^2^ = −1, and *f* is the frequency. The complex permittivity of hydraulic oil and particles are ${\tilde{\varepsilon }}_{o}$ and ${\tilde{\varepsilon }}_{p}$, respectively.

(14) $${\tilde{\varepsilon }}_{o}=\varepsilon_{o}-j\frac{\sigma_{o}}{2\pi f};$$

(15) $${\tilde{\varepsilon }}_{p}=\varepsilon_{p}-j\frac{\sigma_{p}}{2\pi f};$$

where $\sigma_{o}$ is the conductivity of hydraulic oil, and $\sigma_{p}$ is the conductivity of particles in the detection zone. According to Maxwell’s mixture equation, combined with the previous theoretical derivation of our group, the equivalent complex permittivity of the mixture containing the particles and oil is [31]:

(16) $${\tilde{\varepsilon }}_{\mathrm{mix}}={\tilde{\varepsilon }}_{o}\frac{V_{d}\left({\tilde{\varepsilon }}_{p}+2{\tilde{\varepsilon }}_{o}\right)+V_{p}\left({\tilde{\varepsilon }}_{p}-{\tilde{\varepsilon }}_{o}\right)}{V_{d}\left({\tilde{\varepsilon }}_{p}+2{\tilde{\varepsilon }}_{o}\right)-V_{p}\left({\tilde{\varepsilon }}_{p}-{\tilde{\varepsilon }}_{o}\right)},$$

where $V_{p}$ is the particle volume, and $V_{d}$ is the detection volume in the detection zone. Substituting Equation (16) into Equation (12), we can get the expression of the equivalent capacitance of double solenoid coils:

(17) $$C=\frac{\pi \varepsilon_{0}{\tilde{\varepsilon }}_{o}(R^{2}-r^{2})}{r}\left[1+\frac{2V_{p}\left({\tilde{\varepsilon }}_{p}-{\tilde{\varepsilon }}_{o}\right)}{V_{d}\left({\tilde{\varepsilon }}_{p}+2{\tilde{\varepsilon }}_{o}\right)-V_{p}\left({\tilde{\varepsilon }}_{p}-{\tilde{\varepsilon }}_{o}\right)}\right].$$

From Equation (10), capacitance is related to the relative permittivity and the volume of particles when the structural parameters of double solenoid coils and the excitation alternating current are determinate. In detecting the same kind of particles, the larger the particle volume, the larger the capacitance change. Therefore, the amount of capacitance change can directly reflect the size of particles. Since the relative permittivity of air (${\tilde{\varepsilon }}_{p,a}$ ≈ 1) is less than the relative permittivity of oil (${\tilde{\varepsilon }}_{o}$ ≈ 2.6), and the relative permittivity of water (${\tilde{\varepsilon }}_{p,w}$ ≈ 80) is greater than the relative permittivity of oil, the output capacitance will generate a downward and upward pulse signal for detecting air bubbles and water droplets. So, the capacitance detection mode could distinguish between water droplets and air bubbles in oil based on the difference in relative permittivity.

## 3. Chip Fabrication and Experimental Preparation

### 3.1. Chip Fabrication

According to the detection chip fabrication methods proposed by our group [32], a pair of two identical solenoid coils (300 turns, the inner diameter was 0.7 mm, the outer diameter was 1.9 mm, and the axial length was 2.8 mm) were fabricated first as the double solenoid coils model. Then, the same two silicon steel sheets were respectively applied with glue and inserted into the solenoid coils with a tweezer. A copper rod with a diameter of 0.3 mm was used as the straight microchannel model and was fixed on a glass substrate. The double solenoid coils inserted into the silicon steel sheets were fixed on the glass substrate and pressed against the copper rod. After that, the solenoid coils, microchannel model, and glass substrate formed a chip mold, upon which prepared liquid polydimethylsiloxane (the ratio of polydimethylsiloxane to curing agent was 10:1) was poured. The chip mold was then placed in a thermostat with a temperature of 80 °C for 1 h. Finally, after the liquid polydimethylsiloxane was solidified, a copper rod with a diameter of 0.3 mm was extracted from the solidified PDMS using pliers, and fabrication of the multifunctional detection chip was completed. In order to investigate the influence of silicon steel sheets on the detection sensitivity of the design sensor, the double solenoid coils with and without silicon steel sheets were fabricated in a microfluidic detection chip for comparative analysis.

### 3.2. Construction of the Detection System

**Figure 4:**
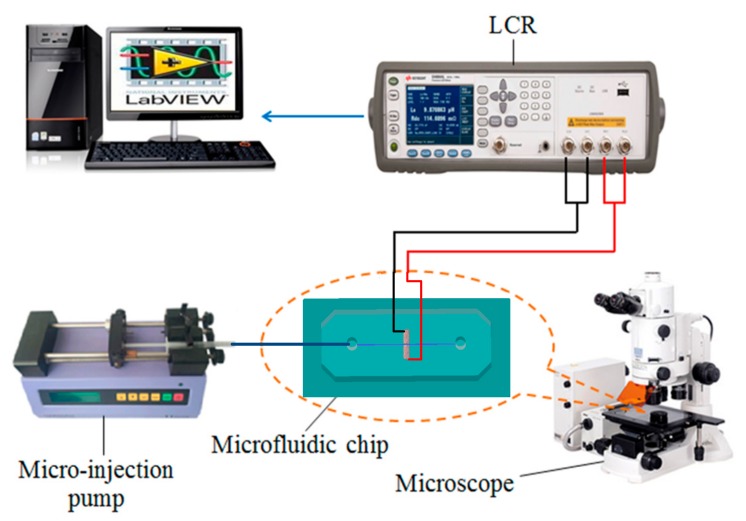
Schematic diagram of the impedance detection system.

After fabrication of the multifunction detection chip, the oil detection system was built as shown in Figure 4. It consisted of a LabVIEW data acquisition unit, an LCR meter (Keysight E4980A, Agilent Technologies Inc., Bayan Lepas, Malaysia), a microinjection pump (Harvard Apparatus B-85259, Harvard Apparatus, Holliston, MA, USA), a microscope (Nikon AZ100, Nikon, Tokyo, Japan), and the multifunction detection chip. The LabVIEW data acquisition unit and the LCR meter could record and display the equivalent inductance or equivalent capacitance of the detection chip coil in real time, and the microscope could be used to help determine whether signal fluctuation was caused by particles.

### 3.3. Sample Preparation 

For inductance detection experiments, it was necessary to prepare hydraulic oil samples containing iron particles and copper particles of different sizes. Firstly, the sizes of iron particles ranging from 60 to 70 μm, 90 to 100 μm, and 120 to 130 μm were sieved with different sieves. Iron particles (5 mg) of each size were weighed with a precision balance, mixed with 100 mL hydraulic oil, and oscillated uniformly with an oscillator. The same process was also used for 5 mg copper particles ranging from 120 to 130 μm, 130 to 140 μm, and 170 to 180 μm. Then, each particle sample of 1 mL was placed in a plastic test tube for the experiments. 

For capacitance detection experiments, an oscillating method was used to prepare the oil samples containing water droplets and air bubbles with different sizes. To prepare 140 to 150 μm water droplets in the hydraulic oil, 5 μL of distilled water and 995 μL of hydraulic oil were first mixed in a 1 mL sealed plastic tube, and then the plastic tube was oscillated by an oscillator and an ultrasonic oscillator for 1 min and 5 min to generate 140 to 150 μm water droplets. Using a similar method to prepare 240 to 250 μm bubbles in the hydraulic oil, 5 μL air and 995 μL hydraulic oil were sealed in a 1 mL plastic tube, and the tube was oscillated in the oscillator for 1 min, then it was oscillated by the ultrasonic oscillator for 1.5 min to generate 240 to 250 μm bubbles. 

## 4. Results and Discussion

### 4.1. Inductance Detection Results and Discussion

**Figure 5:**
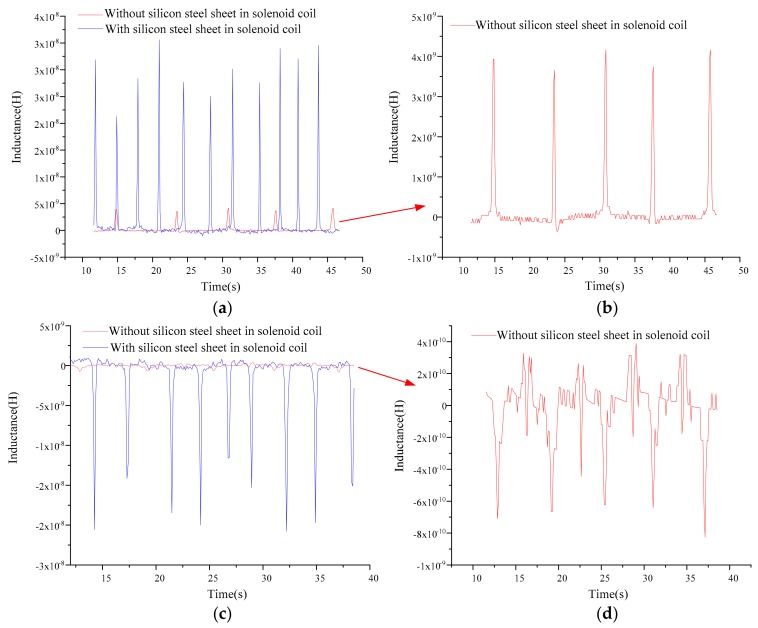
Inductance detection results of 120 to 130 μm iron particles and 170 to 180 μm copper particles: (**a**) iron particle detection results of the solenoid coils with silicon steel sheets; (**b**) iron particle detection results of the solenoid coils without silicon steel sheets; (**c**) copper particle detection results of the solenoid coils with silicon steel sheets; and (**d**) copper particle detection results of the solenoid coils without silicon steel sheets.

In the experiments, the line was connected according to the inductance detection mode, and the excitation voltage and frequency of the LCR meter were set to 2 MHz and 2 V, respectively. The flow rate of the microinjection pump was set to 350 μL/min, then the pump started to inject the sample oil into the multifunction detection chip. A partial detection signal was extracted as shown in Figure 5.

In Figure 5, the directions of the inductance pulses caused by the iron particles and the copper particles were opposite, which verified the inductance detection model. The inductance amplitudes of detecting iron particles and copper particles were extremely improved by inserting the silicon steel sheets into the solenoid coils. For the detection of 120 to 130 μm iron particles, the average inductance amplitude with silicon steel sheets was about 8.6 times that of without silicon steel sheets (the average pulse amplitudes of detecting iron particles with and without silicon steel sheets were about 3.21 × 10^−8^ H and 3.76 × 10^−9^ H, respectively). For the detection of 170 to 180 μm copper particles, the average inductance amplitude of the solenoid coils with silicon steel sheets was 22.7 times that without silicon steel sheets (the average pulse amplitudes of detecting copper particles with and without silicon steel sheets were about −1.98 × 10^−8^ H and −8.73 × 10^−10^ H, respectively). 

**Figure 6:**
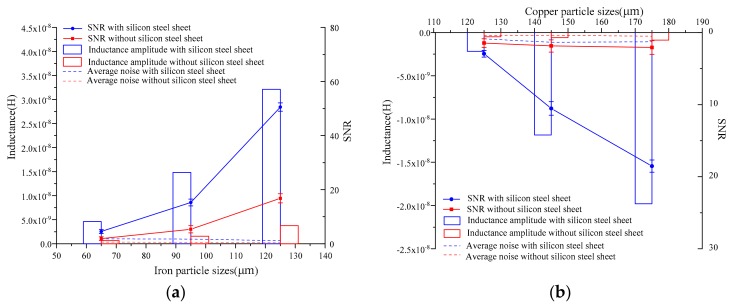
Comparative experimental results of iron particles and copper particles of different sizes: (**a**) iron particle detection comparative results ranging from 60 to 70 μm, 90 to 100 μm, and 120 to 130 μm; (**b**) copper particle detection comparative results ranging from 120 to 130 μm, 130 to 140 μm, and 170 to 180 μm. SNR = signal-to-noise ratio.

In order to more intuitively analyze the influence of silicon steel sheets on detection sensitivity, the inductance pulse amplitude, average noise, and SNR (signal-to-noise ratio) were selected as three sensitivity evaluation indexes. In order to ensure accurate comparisons of the evaluation indexes, the average of the five groups of experimental results was taken as the evaluation index value. The statistical comparison results are shown in Figure 6. 

From Figure 6a, with the increase of iron particle sizes ranging from 60 to 70 μm, 90 to 100 μm, and 120 to 130 μm, the inductance amplitudes of the detection signals were greatly increased by the existence of silicon steel sheets, which were about 7.0, 9.5, and 8.6 times greater, respectively, compared to the solenoid coils without silicon steel sheets. Moreover, existence of silicon steel sheets did not cause a significant increase in noise, so the SNRs were also greatly improved, which were about 2.4, 2.9, and 3.0 times greater, respectively, compared to the solenoid coils without silicon steel sheets. From Figure 6b, with the increase of copper particle sizes ranging from 120 to 130 μm, 130 to 140 μm, and 170 to 180 μm, the inductance amplitude and SNR of the detection signals were also greatly increased by the existence of silicon steel sheets, which were about 4.5, 20.1, and 22.7 and 2.0, 5.7, and 8.9 times greater than those without silicon steel sheets, respectively. Therefore, silicon steel sheets significantly improved the sensitivity of detecting metal particles. As the magnetization of silicon steel sheets was nonlinear, the improvement of detection sensitivity by the silicon steel sheets was also different.

**Figure 7:**
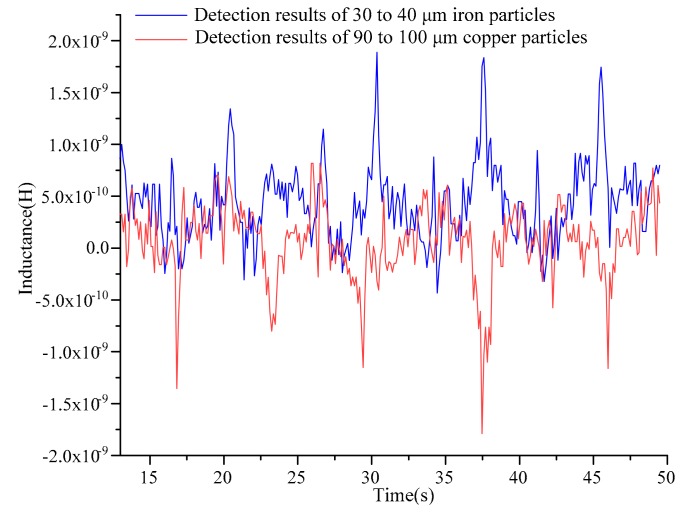
Inductance detection results of 30 to 40 μm iron particles and 90 to 100 μm copper particles with silicon steel sheets.

For the lower limit test of the double solenoid coils with silicon steel sheets, preparation of hydraulic oil samples was the same as described above. The lower limit test was carried out by using iron particles with diameters below 60 μm and copper particles with diameters below 120 μm. The sizes of the particles were gradually reduced in the test, and iron particles with sizes ranging from 30 to 40 µm and copper particles with sizes ranging from 90 to 100 µm were successfully detected. Combining the results of the iron particles test with that of the copper particles test in Figure 7, iron particles ranging from 30 to 40 µm and copper particles ranging from 90 to 100 µm could still be distinguished by the direction of the inductance amplitude.

### 4.2. Capacitance Detection Results and Discussion

**Figure 8:**
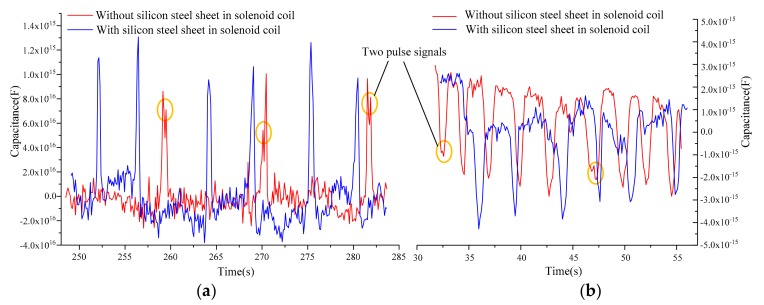
Capacitance detection results of 140 to 150 μm water droplets and 240 to 250 μm bubbles: (**a**) detection results of 140 to150 μm water droplets; (**b**) detection results of 240 to 250 μm bubbles. The red line is the detection results without silicon steel sheets, and the blue line is the detection results with silicon steel sheets.

In the experiments, the line of double solenoid coils was connected according to the capacitance detection mode, and the excitation voltage and frequency of the LCR meter were set to 0.3 MHz and 2 V, respectively. The flow rate of the microinjection pump was set to 350 μL/min, then the pump started to inject the sample oil to the multifunction detection chip. We successfully detected 140 to 150 μm water droplets and 240 to 250 μm bubbles using double solenoid coils with and without silicon steel sheets. The detection signals are shown in Figure 8.

In Figure 8, we can see that the directions of the capacitance pulses caused by the water droplets and bubbles were opposite, which verified the capacitance detection model. At the same time, we found that there were two pulse signals when detecting a single water droplet or bubble using double solenoid coils without silicon steel sheets. This was because the double solenoid coil was equivalent to a circular capacitor, which had two intersections with the microchannel and captured two capacitance pulse signals for the same particle; however, the generation of two pulse signals was also related to particle size and flow velocity. There were no two pulses in the capacitance detection using double solenoid coils with silicon steel sheets. This mainly was due to the additional electric field produced by polarization of silicon steel sheets, which compensated the electric field strength in the inner hole area of double coils. 

Similar to the inductive detection comparison analysis, the capacitance amplitude, average noise, and SNR were selected to evaluate the influence of the silicon steel sheet on the sensitivity of the capacitance detection. The results of the five groups of experiments were statistically analyzed, and the statistical results are shown in Table 1.

In Table 1, the existence of silicon steel sheets in double solenoid coils improved the sensitivity of capacitance detection, but the improvement effect was not obvious with respect to detecting metal particles. The capacitance detection noise was also slightly increased by the silicon steel sheets. The capacitance amplitude and SNR of detecting water droplets increased by 37.4% and 21.9%, respectively, compared to the solenoid coils without silicon steel sheets. The capacitance amplitude and SNR of detecting bubbles increased by 18.5% and 8.0%, respectively, compared to the solenoid coils without silicon steel sheets. 

## 5. Conclusions

In order to enrich the detection ability of the solenoid coil sensor for hydraulic oil contaminants, a multifunction detection sensor based on a microfluidic chip is proposed and fabricated. Firstly, the metal particle inductance detection model and the capacitance detection model of nonmetal particles are theoretically derived. The experimental results show that the sensor can detect iron particles, copper particles, water droplets, and bubbles in hydraulic oil. The detection sensitivity of double solenoid coils by inserting silicon steel sheets is further improved. The inductance (capacitance) amplitude, average noise, and SNR are selected as indicators of sensor sensitivity. The comparative experimental results show that the silicon steels sheets can significantly improve the sensitivity of inductance detection and can improve the sensitivity of capacitance detection, but the improvement effect is not obvious compared to inductance detection. This is probably because the relative permeability of the silicon steel sheet is higher. Compared to the polarization effect, the magnetization effect is dominant in this experiment. We demonstrate the successful detection of 30 μm iron particles and 90 μm copper particles using double solenoid coils with silicon steel sheets as well as the successful detection of 140 μm water droplets and 240 μm bubbles using double solenoid coils with and without silicon steels sheets. Therefore, improving the sensitivity of capacitance detection is our next priority research work. The multifunction sensor presented in the paper integrates the functions of inductance detection and capacitance, and it improves the sensitivity by embedding silicon steel sheets in solenoid coils, which has great significance for health status monitoring and fault diagnosis of hydraulic systems.

## Figures and Tables

**Table 1:** Capacitance detection results of double solenoid coils.

Particles	Chip Structure	Average Amplitude (F)	Average Noise (F)	SNR
water droplets (140–150 μm)	with silicon steel sheets	1.26 × 10^−15^	4.71 × 10^−16^	2.67
	without silicon steel sheets	9.17 × 10^−16^	4.19 × 10^−16^	2.19
Bubbles (240–250 μm)	with silicon steel sheets	4.73 × 10^−15^	1.83 × 10^−16^	2.58
	without silicon steel sheets	3.99 × 10^−15^	1.67 × 10^−16^	2.39
